# A Candidate H1N1 Pandemic Influenza Vaccine Elicits Protective Immunity in Mice

**DOI:** 10.1371/journal.pone.0010492

**Published:** 2010-05-05

**Authors:** Julia Steitz, Peter G. Barlow, Jaber Hossain, Eun Kim, Kaori Okada, Tom Kenniston, Sheri Rea, Ruben O. Donis, Andrea Gambotto

**Affiliations:** 1 Department of Surgery, University of Pittsburgh School of Medicine, Pittsburgh, Pennsylvania, United States of America; 2 Division of Infectious Diseases, Department of Medicine, University of Pittsburgh School of Medicine, Pittsburgh, Pennsylvania, United States of America; 3 Molecular Virology and Vaccines Branch, Influenza Division, National Center for Infectious Diseases, Centers for Disease Control and Prevention, Atlanta, Georgia, United States of America; National Institutes of Health, United States of America

## Abstract

**Background:**

In 2009 a new pandemic disease appeared and spread globally. The recent emergence of the pandemic influenza virus H1N1 first isolated in Mexico and USA raised concerns about vaccine availability. We here report our development of an adenovirus-based influenza H1N1 vaccine tested for immunogenicity and efficacy to confer protection in animal model.

**Methods:**

We generated two adenovirus(Ad5)-based influenza vaccine candidates encoding the wildtype or a codon-optimized hemagglutinin antigen (HA) from the recently emerged swine influenza isolate A/California/04/2009 (H1N1)pdm. After verification of antigen expression, immunogenicity of the vaccine candidates were tested in a mouse model using dose escalations for subcutaneous immunization. Sera of immunized animals were tested in microneutalization and hemagglutination inhibition assays for the presence of HA-specific antibodies. HA-specific T-cells were measured in IFNγ Elispot assays. The efficiency of the influenza vaccine candidates were evaluated in a challenge model by measuring viral titer in lung and nasal turbinate 3 days after inoculation of a homologous H1N1 virus.

**Conclusions/Significance:**

A single immunization resulted in robust cellular and humoral immune response. Remarkably, the intensity of the immune response was substantially enhanced with codon-optimized antigen, indicating the benefit of manipulating the genetic code of HA antigens in the context of recombinant influenza vaccine design. These results highlight the value of advanced technologies in vaccine development and deployment in response to infections with pandemic potential. Our study emphasizes the potential of an adenoviral-based influenza vaccine platform with the benefits of speed of manufacture and efficacy of a single dose immunization.

## Introduction

Events of the past six months have brought home to the world that a new pandemic disease has appeared in our midst. The pandemic caused by a swine-origin influenza virus A H1N1 and first isolated from humans in Mexico and the USA has spread globally [1]–[3]. As of December 30^th^, over 12.220 deaths have been reported associated with this outbreak (http://www.who.int/csr/don/2009_12_30/en/index.html). Through the efforts of an international consortium of laboratories, the swine-origin influenza virus H1N1 was identified in less than 2 months following its first appearance in Mexico and the antigenic and genetic characteristics of 71 isolates have been investigated [4]; [5]. Phylogenetic analysis enabled the identification of the newly emerged swine-origin influenza virus A H1N1 as a reassortant virus evolved from a classical swine H1N1 lineage, an avian-like Eurasian swine H1N1 lineage and from the North America H3N2 triple reassortant influenza lineage [6]; [7]. To prevent and control this unique pandemic swine-origin influenza virus H1N1, both vaccine and antiviral drugs are now available. Antiviral drugs of the neuraminidase inhibitor group have been used effectively against influenza viruses but might not be in sufficient supply and the virus may acquire resistance to the available antiviral drugs [8]. A monovalent influenza virus vaccine has been rapidly produced by standard techniques and is being evaluated in clinical trials [9]. However, it appears that the production of large amounts of egg-produced swine influenza H1N1 vaccine has limited capability due to suboptimal yields (http://www.npr.org/blogs/health/2009/07/manufacturing_problems_with_sw.html). This could severely hinder the ability to produce large amount of vaccines in a timely manner for efficient control of pandemic spread via vaccination.

As human infection with H1N1 viruses continue to increase, so too does the likelihood of antigenic drift and shift leading to generation of novel viruses with potential for higher morbidity and mortality rates and hindering the efficacy of current vaccines. Alternative or complementary vaccine strategies with the feature of speed for manufacture and high yields of vaccine production as well as effective in a single immunization setting would be beneficial.

Recombinant DNA vaccines are highly effective inducers of both humoral and cellular immunity and show promise in the prevention of human disease in animal models [10]–[13]. The successful use of recombinant adenoviruses encoding hemagglutinin from highly pathogenic influenza viruses with pandemic potential has been previously demonstrated [14]; [15]. In this study we tested two adenovirus-based influenza vaccine candidates in an animal model to induce effective H1N1 swine influenza specific immune responses combined with protection.

## Results

We generated E1/E3-deleted adenoviral (Ad5)-based vectors encoding the wildtype (AdHA.wt) or a codon-optimized (AdHA.cod) hemagglutinin (HA) gene from A/California/04/2009 (H1N1)pdm influenza virus. Codon-optimization using the UpGene algorithm improves expression levels of the antigen in infected mammalian cells and consequently enhances immune responses against the antigen *in vivo* [16]. The generation of the recombinant adenoviral vectors was completed 14 days (AdHA.wt) and 21 days (AdHA.cod) after acquisition of the A/California/04/2009 (H1N1)pdm HA sequence, illustrating the rapid construction and ease of manipulation necessary for adenovirus-based vaccine development.

**Figure 1:**
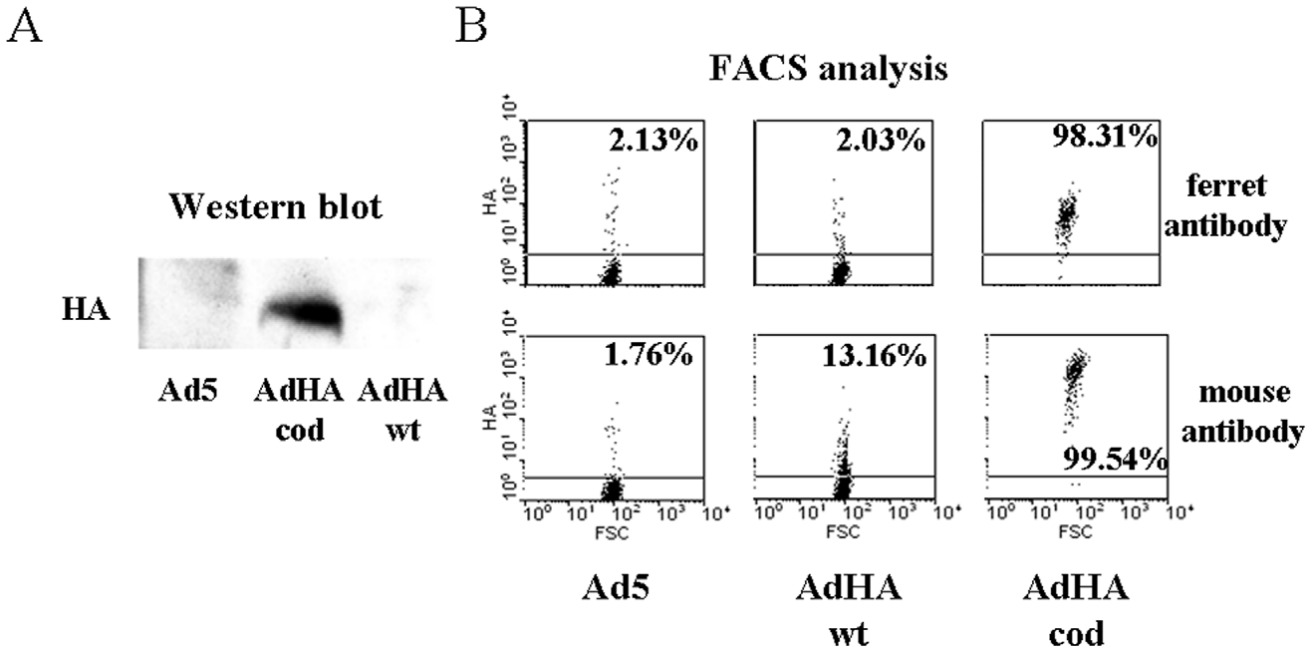
Verification of gene expression by the H1N1 vaccine candidates. HA expression in A549 cells transduced with AdHA.wt, AdHA.cod or Ad5. **A,** HA detection in cell lysates by western blot analysis (60 ug of total protein loaded in each lane) using ferret antiserum. **B**, Flow cytometric analysis of HA expression at the cell surface using ferret or mouse antiserum. Shown are the percentages of HA positive cells.

Initially, the recombinant adenoviral vaccines were evaluated in A549 cells where HA antigen expression levels after infection were examined by immunoblot (Fig. 1A) and flow cytometry (Fig. 1B) analysis using ferret and mouse antiserum. Notably, poor HA expression was seen in cells infected with AdHA.wt, whereas stronger HA expression was detected after infection with AdHA.cod.

**Figure 2:**
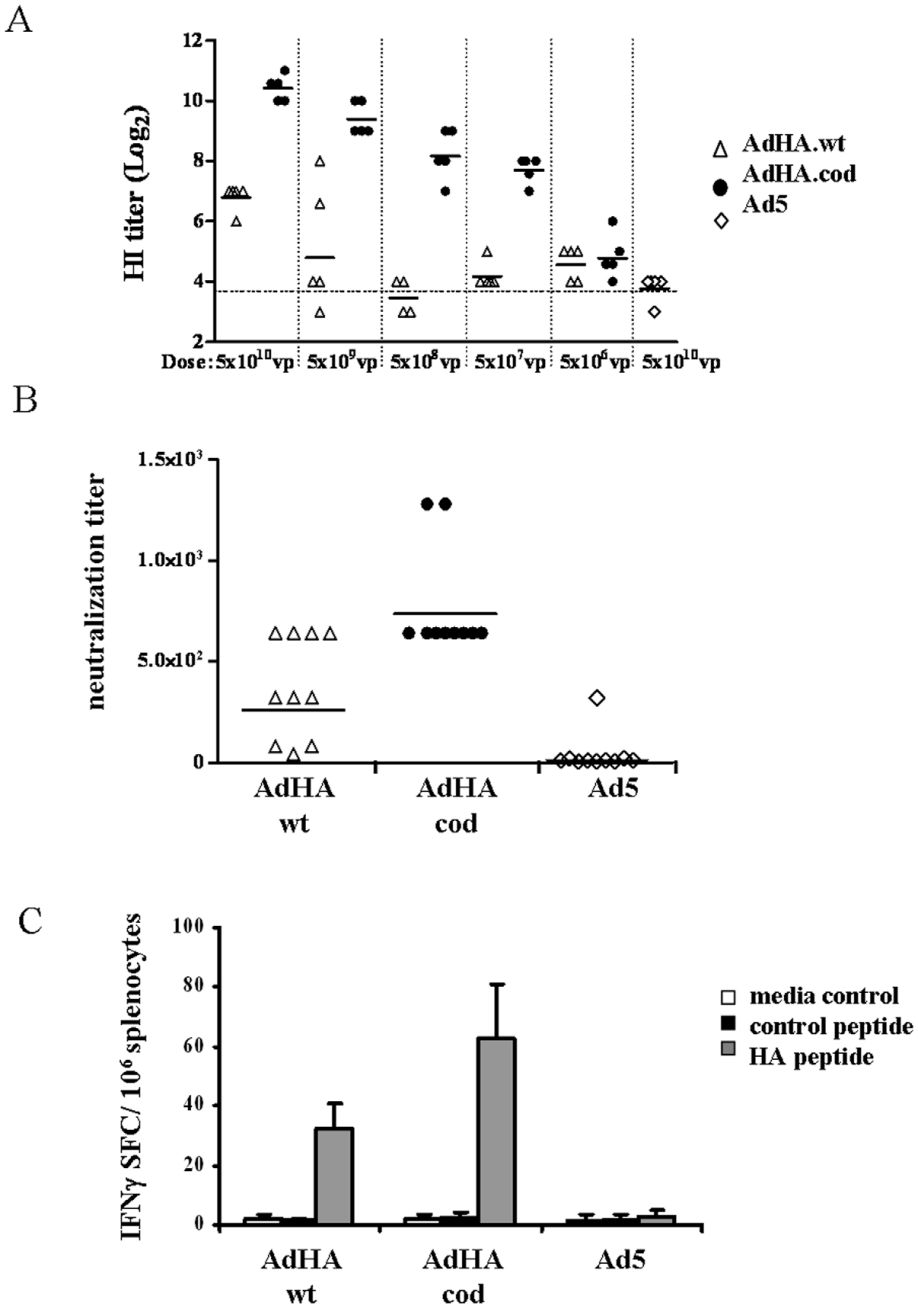
Induction of humoral and cellular immune responses by the H1N1 vaccine candidates. Induction of influenza H1N1-specific immune responses after subcutaneous immunization of Balb/c mice with adenovirus encoding wildtype (AdHA.wt) or codon-optimized (AdHA.cod) HA antigen or control adenovirus (Ad5). **A,** Antibody to influenza A/Texas/05/09 (H1N1)pdm in sera of mice after receiving dose escalations of the vaccine candidates (AdHA.wt or AdHA.cod). Shown are log2 values of HI-titer. Horizontal lines represent geometric means in each group. **B**, Induction of H1N1-specific neutralizing antibodies in sera 5 weeks after immunization with AdHA.wt, AdHA.cod or Ad5 control. Shown are neutralizing antibody titer to influenza A/Texas/05/09 (H1N1)pdm measured by microneutralization assay. Horizontal lines represent geometric means in each group. **C,** HA peptide specific T cell responses in splenocytes detected in IFNγ Elispot assays. A representative result is shown as means of SFC ± SEM of triplicate determinations in each group.

To compare the ability of the two AdHA vaccine candidates to induce HA-specific immune responses, eleven groups of Balb/c mice were immunized subcutaneously with increasing doses of AdHA.wt, AdHA.cod or with Ad5 control. Four weeks after immunization serum samples were obtained from all mice to screen for H1N1 specific antibodies in hemagglutination inhibition (HI) assay using the influenza strain A/Texas/05/09 (H1N1)pdm as source of homologous antigen. HA-specific antibodies response was detected in the animals immunized with AdHA.wt and AdHA.cod. Remarkably, AdHA.cod immunization required at least three log10 less vaccine to achieve the same level of HI immunity measured in the AdHA.wt groups (Fig. 2A). Given that vaccination with AdHA.cod and AdHA.wt induced various degrees of humoral immunity we then only used the highest vaccine dose (5×10^10^ vp/mouse) for immunization for further characterization of the AdHA.wt, AdHA.cod vaccine candidates. To determine the degree to which antibody responses could neutralize homologous A/Texas/05/09 (H1N1)pdm influenza virus, indicating the potential of protection, sera were tested in microneutralization assay (Fig. 2B). Neutralization titers, measured at week 5 after immunization, of ≥640 were observed in AdHA.cod and in some AdHA.wt immunized animals, again with a significant increase in geometric means of neutralization titers observed after AdHA.cod immunization. To determine T-cell specific responses to HA by immunized mice, ELISPOT assays measuring IFN-γ secretion were performed on splenocytes collected at week 5 post vaccination using a previously described heterosubtypic conserved peptide encoded in the HA2 portion of HA antigen [14] as the target T-cell epitope. Both vaccine candidates, AdHA.wt and AdHA.cod, were able to induce HA-peptide-specific T-cell responses (Fig. 2C). However, as seen with antibody responses, the AdHA.cod vaccine induced a greater T-cell specific response.

**Figure 3:**
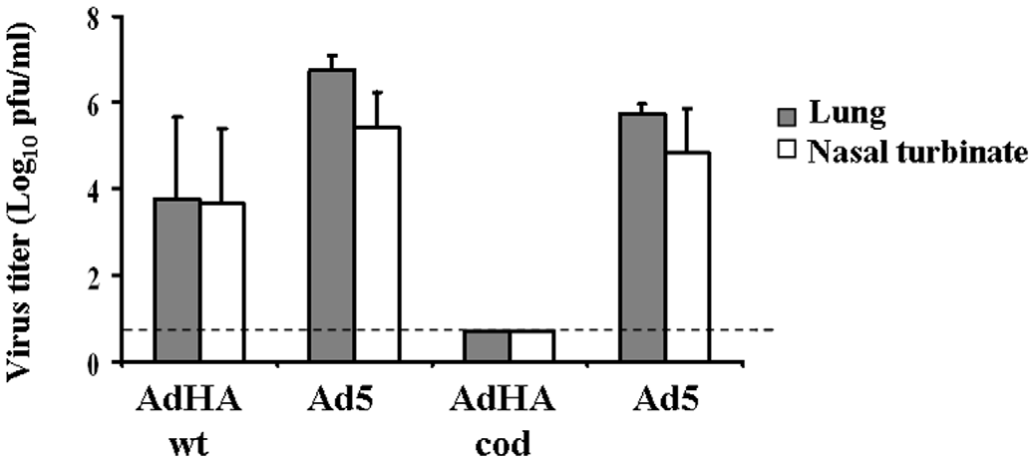
Induced protection against H1N1 by the H1N1 vaccine candidates. Protection of immunized animals (5 or 6 weeks after immunization with AdHA.wt vs Ad5 control or AdHA.cod vs Ad5 control) against an intranasal challenge with 1000 pfu of A/Ohio/7/09 (H1N1)pdm was measured as viral titers in lung and nasal turbinate determined 3 days post-inoculation in a plaque formation assay using MDCK-L cells. Shown are log10 values of mean titer for each group ± SEM. The horizontal dashed line represents the lower limit of detection of the assay.

To investigate the protective efficacy of these AdHA vaccine candidates against challenge with the H1N1 virus we utilized a mouse model [17]. Animals were intranasally inoculated with 1000 PFU A/Ohio/7/09 (H1N1)pdm 5 to 6 weeks after the single dose immunization. Three days post-challenge, the mice were sacrificed, their lungs and nasal turbinate harvested and viral titers were determined by plaque formation assay performed in MDCK-L cells. As expected, the mock-immunized group had positive H1N1 titer in lung and nasal turbinate. No measurable virus titers were detected in lung and nasal turbinate in the AdHA.cod immunized group (Fig. 3). Somewhat lower levels of protection were observed in the AdHA.wt immunized group. As shown in table 1, in AdHA.wt immunized group 2 out of 10 animals had positive H1N1 titer revealing a correlation between the magnitude of the immune response and protection from H1N1 challenge.

**Table 1:** Protection against influenza A/Ohio/7/09 (H1N1)pdm.

Immunization	Virus in Lung(1)	Virus in Nasal Turbinate (1)
AdHAwt	2/10	8/10
Ad5 control	5/5	5/5
AdHAcod	0/10	0/10
Ad5 control	5/5	5/5

(1) number of animals from which virus was cultured/number of animals inoculated.

## Discussion

This study demonstrates that a single-dose of codon-optimized adenovirus-based H1N1 vaccine expressing HA derived from influenza virus A/California/04/2009 (H1N1)pdm efficiently induces HA-specific antibodies and T-cells in mice that correlates with protection when measured as the elimination of detectable virus titers in lungs and nasal turbinates 3 days after challenge. The intensity of the immune response was greater in the group of mice immunized with AdHA.cod, indicating a benefit associated with optimizing the genetic code of antigen-expressing genes in recombinant vaccines as observed in other studies [18]–[20]. Though the correlation of induction of HA-specific antibodies to vaccine doses was clearly demonstrated, we did not measure T-cell frequencies following immunization with different vaccine doses. Thus, future studies to determine the impact of T cell frequency and the correlation with HA antigen expression levels and protection would be of great interest.

These findings can be extended to other genetically engineered H1N1 influenza vaccines using reverse genetic techniques [21]; [22] or using other recombinant viral vectors [23]–[25], and warrant the use of a codon-optimized version of the gene encoding HA. Enhancing the HA expression in the reassortant influenza vaccine strain could result in higher yields of the HA protein purified from the allantoic fluid of virus infected chicken eggs and consequently more vaccine doses per batch.

The use of recombinant adenoviral vectors to immunize against infectious diseases and cancer has been proven safe in more than 100 phase I, II, and III clinical trials. The effective use of adenoviral-based influenza HA vaccines was demonstrated in previous studies in mice, chickens and humans [14]; [15]; [26] showing promising results indicating the adenoviral platform as a safe and practicable alternative to propagating vaccines with conventional methods in embryonated chicken eggs. Moreover, the ability to produce the adenoviral seed stock generation only 2–3 weeks after the appearance of the newly emerged influenza strain is one of the most valuable properties of this vaccine technology. However, natural pre-existing immunity against adenoviral vectors [27] could potentially reduce vaccine efficacy. While the impact of natural neutralizing antibodies on the induction of effective immune responses is still controversial, the effective delivery of an adenovirus-based influenza HA vaccine via the mucosal route has been demonstrated, by van Kampen et al and our own results (data not shown), effective despite the presence of anti-adenoviral antibodies, suggesting that vector-specific immunity may be overcome [26]. Extensive *in vivo* work comparing different routes of administration as a way to overcome pre-existing adenoviral neutralizing immunity is underway in our laboratory and will be the subject of future manuscripts.

Alternatively, a wide range of different human and animal adenovirus serotypes, as well as structural and nonstructural modifications of adenoviral vectors, have been investigated to circumvent pre-existing adenoviral immunity [28]–[31].

In conclusion, this study further emphasizes the vaccine potential of an adenoviral-based platform with the advantages of speed for the seed stock generation and high yields of vaccine virus in a large-scale production. Furthermore, this vaccine elicits robust cellular and humoral immunity after a single dose immunization. These properties are invaluable when rapid vaccine development and production are required to control the virus spread and mitigate the public health impact of pandemic influenza. Although still experimental, these results mandate further testing of adenovirus-based influenza vaccine technology in human clinical trials.

## Materials and Methods

### Ethics Statement

All animals were housed and handled according to the University of Pittsburgh's Institutional Animal Care and Use Committee guidelines and all animal work was approved by the appropriate committee (IACUC 0909547 and 0802046A-1).

### Viruses and Cells

A549, a human lung adenocarcinoma epithelial cell line, cells were obtained from ATCC. A/California/04/2009, A/Texas/05/09, A/Ohio/7/09 pandemic H1N1, (H1N1)pdm, influenza viruses were obtained from the Influenza Reagent Resource or the WHO Influenza Collaborating Center in Atlanta. The three viruses are antigenically identical to A/California/07/2009 (H1N1)pdm, which was recommended for vaccine development by the WHO. Different pandemic virus strains were used in HI, microneutralization and challenge assays according to their availability.

### Gene synthesis and adenoviral vector constructions

Hemagglutinin HA gene (GenBank Accession FJ966082) was PCR amplified from influenza virus A/California/04/2009 followed by cloning into pCI vector. Alternatively the HA gene was codon-optimized for optimal expression in mammalian cells by the UpGene codon optimization algorithm (http://www.vectorcore.pitt.edu/upgene.html) and synthesized by GenScript. Both genes were subcloned into pAdlox (GenBank U62024) to generate recombinant adenoviruses of the serotype 5 as previously described [14]; [32]. Generation of the wild type HA-encoding adenovirus (AdHA.wt) required 14 days. Conversely the generation of the codon-optimized HA-encoding adenovirus, due to the extra time required for the gene synthesis, required 21 days. All vaccine candidates were controlled by sequencing.

### HA expression analysis

A549 cells were seeded at 6-well plates and infected with AdHA.wt, AdHA.cod or Ad5 (10^10^ vp/well). After 24 h at 37°C, cells were harvested, trypsinized, washed with PBS and stained with ferret antiserum against A/Texas/05/09 (H1N1)pdm or mouse antiserum against AdHAcod followed by a FITC-conjugated anti-ferret or PE-conjugated anti-mouse secondary antibody. Data acquisition and analysis was performed using a FACScan and CELLQuest software. Alternatively, cell lysates of AdHA-infected A549 cells were tested for HA expression in western blot analysis using ferret antiserum (against A/Texas/05/09 (H1N1)pdm).

### Animal studies

Female Balb/c mice (five mice per group) were immunized via subcutaneous inoculation of 5×10^10^, 5×10^9^, 5×10^8^, 5×10^7^ or 5×10^6^ virus particles each of AdHA.wt, AdHA.cod and 5×10^10^ control Ad5 virus. Four weeks later immune sera and splenocytes were harvested and further tested in HI assay and IFNγ Elispot. For challenge studies ten mice per group were immunized via subcutaneous inoculation of 5×10^10^ virus particles each of AdHA.wt or control Ad5 virus. Due to delayed availability of the codon-optimized gene, groups of animals received 5×10^10^ vp of AdHA.cod vaccine or control Ad5 one week later. All groups were intranasally challenged at the same time with a non-lethal dose (1000 PFU) of influenza A/Ohio/7/09 (H1N1)pdm in 50 µl of PBS. At 3 days after inoculation lungs and nasal turbinates were collected for virus quantification.

### Hemagglutination inhibition test

Four weeks after immunization, sera were collected and treated with receptor destroying enzyme (RDE) (Denka-Seiken) and subsequently tested in a standard hemagglutination inhibition test using 4 HAU of influenza A/Texas/05/09 (H1N1)pdm virus as source of antigen and 0.5% turkey red blood cell solution in PBS [14].

### Microneutralization assay

Serum neutralizing antibody titers were determined by seeding MDCK cells at 1.5×10^4^ cells/well in 96-well culture plates and culturing at 37°C in 5% CO_2_ to form a monolayer. Serial two fold dilutions of heat inactivated serum samples were mixed separately with 100 TCID_50_ of A/Texas/05/09 (H1N1)pdm and incubated at room temperature for 2 h. The mixture was added to monolayer of MDCK cells in triplicate wells. The plates were incubated for 18 h at 37°C and 5% CO_2_.The monolayers were washed with PBS and fixed in cold 80% acetone for 10 min. The presence of viral protein was detected by ELISA with a monoclonal antibody to the influenza A NP and endpoint titer were determined as described previously [33].

### ELISPOT assay for IFNγ

96-well Elispot plates (MAHA S4510, Millipore) were incubated with 10 ug/ml of monoclonal antibody to mouse IFNγ (cloneR46A2, eBioscience) in PBS. Splenocytes were plated at 10^6^ cells per well in medium containing 10% FBS and stimulated with 10 ug/ml of the previously described HA2 peptide IYQILSIYSTVASSL [14]. This hetrosubtypic peptide was used due to availability and homology to the HA2 peptide encoded by H1N1 IYQILAIYSTVASSL. After incubation at 37°C for 24 h, wells were washed and incubated with biotinylated antibody to mouse IFN-γ (clone XMG 1.2, eBioscience) overnight at at 4°C. After washing with tap water and 1 h incubation with streptavidin-AP conjugate (Dako) at room temperature spots were developed using alkaline phosphatase substrate BCIP/NBT (Sigma) and enumerated using a dissecting microscope.

### Viral lung titer measurement

Three days post infection with influenza virus A/Ohio/7/09(H1N1), mice were sacrificed and lungs and nasal turbinates were collected and frozen at −80°C. Tissue homogenates were prepared by mechanical disruption and used to determine viral titers by plaque formation assay performed in MDCK-L cells in the presence of trypsin [34].

## References

[1] Dawood FS, Jain S, Finelli L, Shaw MW, Lindstrom S (2009). Emergence of a novel swine-origin influenza A (H1N1) virus in humans.. N Engl J Med.

[2] Butler D (2009). Swine flu goes global.. Nature.

[3] Cohen J, Enserink M (2009). Infectious diseases. As swine flu circles globe, scientists grapple with basic questions.. Science.

[4] Garten RJ, Davis CT, Russell CA, Shu B, Lindstrom S (2009). Antigenic and Genetic Characteristics of Swine-Origin 2009 A(H1N1) Influenza Viruses Circulating in Humans.. Science.

[5] Smith GJ, Vijaykrishna D, Bahl J, Lycett SJ, Worobey M (2009). Origins and evolutionary genomics of the 2009 swine-origin H1N1 influenza A epidemic.. Nature.

[6] Peiris JS, Poon LL, Guan Y (2009). Emergence of a novel swine-origin influenza A virus (S-OIV) H1N1 virus in humans.. J Clin Virol.

[7] Neumann G, Noda T, Kawaoka Y (2009). Emergence and pandemic potential of swine-origin H1N1 influenza virus.. Nature.

[8] Poland GA, Jacobson RM, Ovsyannikova IG (2009). Influenza virus resistance to antiviral agents: a plea for rational use.. Clin Infect Dis.

[9] Greenberg ME, Lai MH, Hartel GF, Wichems CH, Gittleson C (2009). Response after one Dose of a Monovalent Influenza A (H1N1) 2009 Vaccine.. N Engl J Med epub ahead of print.

[10] Barouch DH, Santra S, Schmitz JE, Kuroda MJ, Fu TM (2000). Control of viremia and prevention of clinical AIDS in rhesus monkeys by cytokine-augmented DNA vaccination.. Science.

[11] Barouch DH, Craiu A, Santra S, Egan MA, Schmitz JE (2001). Elicitation of high-frequency cytotoxic T-lymphocyte responses against both dominant and subdominant simian-human immunodeficiency virus epitopes by DNA vaccination of rhesus monkeys.. J Virol.

[12] Chen JD, Yang Q, Yang AG, Marasco WA, Chen SY (1996). Intra- and extracellular immunization against HIV-1 infection with lymphocytes transduced with an AAV vector expressing a human anti-gp120 antibody.. Hum Gene Ther.

[13] Shiver JW, Fu TM, Chen L, Casimiro DR, Davies ME (2002). Replication-incompetent adenoviral vaccine vector elicits effective anti-immunodeficiency-virus immunity.. Nature.

[14] Gao W, Soloff AC, Lu X, Montecalvo A, Nguyen DC (2006). Protection of mice and poultry from lethal H5N1 avian influenza virus through adenovirus-based immunization.. J Virol.

[15] Hoelscher MA, Garg S, Bangari DS, Belser JA, Lu X (2006). Development of adenoviral-vector-based pandemic influenza vaccine against antigenically distinct human H5N1 strains in mice.. Lancet.

[16] Gao W, Rzewski A, Sun H, Robbins PD, Gambotto A (2004). UpGene: Application of a web-based DNA codon optimization algorithm.. Biotechnol Prog.

[17] Itoh Y, Shinya K, Kiso M, Watanabe T, Sakoda Y (2009). In vitro and in vivo characterization of new swine-origin H1N1 influenza viruses.. Nature.

[18] Ramakrishna L, Anand KK, Mahalingam M, Mohankumar KM, Ramani S (2004). Codon optimization and ubiquitin conjugation of human immunodeficiency virus-1 Tat lead to enhanced cell-mediated immune responses.. Vaccine.

[19] Mossadegh N, Gissmann L, Muller M, Zentgraf H, Alonso A (2004). Codon optimization of the human papillomavirus 11 (HPV 11) L1 gene leads to increased gene expression and formation of virus-like particles in mammalian epithelial cells.. Virology.

[20] Ternette N, Tippler B, Uberla K, Grunwald T (2007). Immunogenicity and efficacy of codon optimized DNA vaccines encoding the F-protein of respiratory syncytial virus.. Vaccine.

[21] Lipatov AS, Webby RJ, Govorkova EA, Krauss S, Webster RG (2005). Efficacy of H5 influenza vaccines produced by reverse genetics in a lethal mouse model.. J Infect Dis.

[22] Ozawa M, Goto H, Horimoto T, Kawaoka Y (2007). An adenovirus vector-mediated reverse genetics system for influenza A virus generation.. J Virol.

[23] Mayrhofer J, Coulibaly S, Hessel A, Holzer GW, Schwendinger M (2009). Nonreplicating vaccinia virus vectors expressing the H5 influenza virus hemagglutinin produced in modified Vero cells induce robust protection.. J Virol.

[24] DiNapoli JM, Yang L, Suguitan A, Elankumaran S, Dorward DW (2007). Immunization of primates with a Newcastle disease virus-vectored vaccine via the respiratory tract induces a high titer of serum neutralizing antibodies against highly pathogenic avian influenza virus.. J Virol.

[25] Schwartz JA, Buonocore L, Roberts A, Suguitan A, Kobasa D (2007). Vesicular stomatitis virus vectors expressing avian influenza H5 HA induce cross-neutralizing antibodies and long-term protection.. Virology.

[26] Van Kampen KR, Shi Z, Gao P, Zhang J, Foster KW (2005). Safety and immunogenicity of adenovirus-vectored nasal and epicutaneous influenza vaccines in humans.. Vaccine.

[27] Nwanegbo E, Vardas E, Gao W, Whittle H, Sun H (2004). Prevalence of neutralizing antibodies to adenoviral serotypes 5 and 35 in the adult populations of The Gambia, South Africa, and the United States.. Clin Diagn Lab Immunol.

[28] Lasaro MO, Ertl HC (2009). New insights on adenovirus as vaccine vectors.. Mol Ther.

[29] Farina SF, Gao GP, Xiang ZQ, Rux JJ, Burnett RM (2001). Replication-defective vector based on a chimpanzee adenovirus.. J Virol.

[30] Gao W, Robbins PD, Gambotto A (2003). Human adenovirus type 35: nucleotide sequence and vector development.. Gene Ther.

[31] Singh N, Pandey A, Jayashankar L, Mittal SK (2008). Bovine adenoviral vector-based H5N1 influenza vaccine overcomes exceptionally high levels of pre-existing immunity against human adenovirus.. Mol Ther.

[32] Hardy S, Kitamura M, Harris-Stansil T, Dai Y, Phipps ML (1997). Construction of adenovirus vectors through Cre-lox recombination.. J Virol.

[33] Rowe T, Abernathy RA, Hu-Primmer J, Thompson WW, Lu X (1999). Detection of antibody to avian influenza A (H5N1) virus in human serum by using a combination of serologic assays.. J Clin Microbiol.

[34] Szretter KJ, Balish AL, Katz JM (2006). Influenza: propagation, quantification, and storage.. Curr Protoc Microbiol Chapter.

